# Mechanisms of action of tasquinimod on the tumour microenvironment

**DOI:** 10.1007/s00280-013-2321-8

**Published:** 2013-10-27

**Authors:** E. Raymond, A. Dalgleish, J.-E. Damber, M. Smith, R. Pili

**Affiliations:** 1 Department of Medical Oncology, Beaujon University Hospital, Clichy, France; 2 Division of Cellular and Molecular Medicine, Department of Oncology, St George’s, University of London, London, UK; 3 Department of Urology, Institute of Clinical Sciences, Gothenburg University, Göteborg, Sweden; 4 Massachusetts General Hospital Cancer Center, Boston, USA; 5 Roswell Park Cancer Institute, Buffalo, NY USA; 6 Service de Cancérologie, Hôpital Beaujon, 100 Bld du Général Leclerc, 92110 Clichy, France

**Keywords:** Tasquinimod, Quinoline carboxamide, Tumour microenvironment, Myeloid-derived suppressor cell, Tumour-associated macrophage, S100A9

## Abstract

Tasquinimod is a small molecule with pleiotropic effects on the tumour microenvironment. Tasquinimod inhibits the growth and metastasis of tumour cells in vitro and in vivo. It targets the tumour microenvironment, enhancing the host immune response and inhibiting the angiogenic response. Tasquinimod influences infiltrating myeloid cells in the tumour milieu shifting the balance towards a less immunosuppressive phenotype. Myeloid-derived suppressor cells and tumour-associated macrophages are major components of the immunosuppressive microenvironment and as a result promote tumour growth and favour angiogenesis and metastasis formation. Growing evidence indicates that tasquinimod targets these myeloid cells and modulates local tumour immunity by blocking the interaction between the multifunctional protein S100A9 and its ligands receptor of advanced glycation end products and Toll-like receptor 4. Its anti-angiogenic effects are achieved at least in part through these effects on regulatory myeloid cells and also potentially through inactivating histone deacetylase-4 and reducing expression of hypoxia-inducible factor 1-controlled genes. The aim is to comprehensively review the mode of action of tasquinimod as a novel oral anti-cancer agent. Based on its unique combination of effects, tasquinimod is a novel agent with clinical therapeutic potential in various solid tumours, both alone and as part of rational combination therapy.

## Introduction

The tumour microenvironment plays a key role in supporting the growth, invasion and metastasis of malignant tumour cells and in protecting the cancer cells from the host immune response [1]. Stromal cells in the microenvironment have a powerful influence on cancer development, and full manifestation of the malignant characteristics of cancer cells depends on complex interactions between cancer cells and the surrounding stroma cells, including immune cells, angiogenic vascular cells and cancer-associated fibroblasts. Interactions of cancer cells with cellular and non-cellular components of the microenvironment occur through various membrane receptors and specialised proteins that can bind to matrix collagen, providing important signals for cancer growth and invasion. Changes in the microenvironment of cancer cells also influence the development of mechanisms by which tumour cells are able to proliferate and metastasise. Although the intrinsic aggressiveness of cancer cells is initiated by oncogenic mutations in major oncogenes, their adaptation to the stroma is likely to be regulated by several epigenetic factors that facilitate survival and invasion [2]. This plasticity of tumour cells to adapt to the surrounding environment has been recognised in a number of preclinical models. Among the most obvious changes are epigenetic modifications in genomic expression and phenotypic modifications that drive epithelial-to-mesenchymal transition. Hence, the tumour milieu represents a critical target for intervention, with increasing interest in the potential for novel therapeutic and prevention strategies to act on the surroundings rather than the tumour itself [3–5].

Tasquinimod is a novel oral quinoline-3-carboxamide derivative with multiple effects on the tumour microenvironment, which is currently at an advanced stage of clinical evaluation as an anti-cancer agent. Among other clinical trials, tasquinimod is currently being studied in a Phase III trial in metastatic prostate cancer and in a Phase II trial in hepatocellular, ovarian, gastric and renal cell carcinomas (clinicaltrials.gov, NCT01234311, NCT01743469). The aim of this review is to summarise the available data and provide an overview of the biological properties of tasquinimod that contribute to its anti-tumour effects.

## The tumour microenvironment

### Role of immune system in promoting tumour growth

Immunotherapy for cancer uses the potential of the host immune response to recognise and eliminate tumour cells. Modulation of the immune response has been the subject of intensive preclinical and clinical research to control tumour growth. The innate and adaptive immune systems can mediate anti-tumour immunity; however, as tumours progress, they escape from immune surveillance through various mechanisms [6]. Among the factors suppressing the immune response to cancer cells, myeloid cells with pro-inflammatory and immunosuppressive effects, namely myeloid-derived suppressor cells (MDSC) and tumour-associated macrophages (TAM), have been the focus of specific attention [7].

The association between chronic inflammation and cancer is well recognised; prolonged presence of an inflammatory milieu predisposes to an increased risk for developing cancer and facilitates tumour development and progression [4, 8, 9]. Chronic inflammation is a complex process that promotes carcinogenesis and tumour progression, although the mechanisms by which specific inflammatory mediators contribute to tumour growth are not fully understood. Inflammatory mediators induce the accumulation of myeloid cells, including MDSC and TAM, which are strongly immunosuppressive and can be found in most models of solid tumours and in clinical cancer [7, 10, 11].

TAM associated with tumours are predominantly the M2 phenotype, which suppress adaptive immunity, encourage angiogenesis and support metastasis through the expression of cytokines, growth factors and matrix metalloproteases [7], in contrast to the classically activated M1 phenotype which promote immune responses and inhibit angiogenesis. MDSC are a heterogeneous population of early myeloid progenitors defined by function and characterised in mice by the expression of CD11b and Gr-1. They mediate local tumour immunity and facilitate carcinogenesis and tumour growth and progression by inhibiting T and NK (natural killer) cell activation and also directly stimulate tumour growth and metastasis by encouraging angiogenesis and creating a favourable environment for metastasis formation [12, 13].

Suppression of tumour-specific T cell sensitisation is an important mechanism that results in uncontrolled proliferation of cancer cells. MDSC present in the tumour microenvironment have the capacity to suppress the cytotoxic effects of NK cells and the adaptive immune response mediated by CD4+ and CD8+ T cells, strongly inhibiting the anti-tumour effects of these cells [14, 15]. Hence, MDSC are thought to play a key role in promoting tumour-associated immune suppression and allowing tumour growth and are thus a potential target for preventing tumour progression. Accordingly, targeting MDSC in the tumour microenvironment is considered a promising therapeutic strategy.

### Role of tumour angiogenesis

The term angiogenesis describes the development and growth of new capillary blood vessels from existing vessels, and this is an essential step necessary to supply oxygen and nutrients to tumours once they are larger than a few millimetres. It has long been recognised that blockade of angiogenesis is a potential anti-cancer intervention, since solid tumours are dependent on this process for sustained growth and metastasis [16]. Hence, inhibition of tumour angiogenesis, through inhibition of the vascular endothelial growth factor (VEGF) signalling pathway, has been actively pursued as a promising therapeutic strategy [17].

Angiogenesis is a complex process regulated by the highly coordinated function of various proteins with pro- and anti-angiogenic functions [18, 19]. As a tumour increases in size and outgrows the existing blood supply, hypoxia drives the angiogenic response (angiogenic switch) through the hypoxia-inducible factor (HIF) pathway. The hypoxic tumour microenvironment contributes to tumour progression by activating a series of adaptive responses that include tumour neovascularisation and tumour-specific immune responses, mediated through the key transcriptional regulators HIF-1α and HIF-2α. HIF activity is regulated by an oxygen-dependent mechanism, and in hypoxia, the HIF-1α subunit is stabilised, moves to the nucleus, forms a dimer with the HIF-1β subunit and binds to its co-activator, p300. This complex regulates expression of hypoxia-responsive genes, including those involved in angiogenesis (and many others) [20].

## Anti-tumour effects of tasquinimod in preclinical models

A series of quinoline-3-carboxamide analogues were identified in the early 1980s, and the lead molecule, linomide, was found to stimulate the immune response and inhibit the growth and metastasis of cancer cells in preclinical models. This compound was evaluated in clinical trials, but development was stopped because of unacceptable toxicity, attributed to dose-dependent pro-inflammatory reactions [21]. Tasquinimod was subsequently selected for development (from a library of compounds) on the basis of its low potential for pro-inflammatory side effects and much higher potency (30–60-fold more potent) than linomide [21–23]. It entered Phase I trials in 2005 [21].

The anti-tumour effects of tasquinimod have been documented in a wide range of preclinical models including both rodent tumours and human tumour xenografts (Table 1) [22, 24, 25]. The activity of tasquinimod against human prostate cancer was evaluated using xenografts in intact male nude mice chosen to encompass the range of phenotypes and genotypes typical of clinical tumours in patients: localised and metastatic, androgen receptor (AR)-positive and AR-negative, AR wild type and mutant, and PSA-positive and PSA-negative cancers. In all five models (CWR-22RV1, LAPC-4, LNCaP, PC-3 and DU-145), tasquinimod treatment at a dose of 1 mg/kg/day for 1 month decreased the tumour volume by at least 50 % (*p* < 0.05) [24]. In subsequent studies, tasquinimod was shown to enhance the efficacy of radiation against human endothelial and prostate cancer cells in culture and human prostate cancer xenografts growing in castrated male nude mice [25]. These in vivo effects were most pronounced when tasquinimod was administered after completion of radiation therapy, thus indicating that tasquinimod interferes with tumour rescue mechanisms [25].

**Table 1:** Preclinical tumour models in which tasquinimod has shown activity [22, 24, 25]

Rodent tumours	Human tumours
EL4 mouse lymphoma Dunning 3327 AT-1 rat prostate cancer TC-2 mouse (TRAMP) prostate cancer	CWR-22Rv1 human prostate cancer (castration-resistant) CWR-22RH human prostate cancer (castration-resistant) LAPC4 human prostate cancer (androgen-sensitive) LNCaP human prostate cancer PC-3 human prostate cancer DU145 human prostate cancer

In addition to inhibiting the growth of primary tumours, tasquinimod prevents metastasis formation in animal models. In a preclinical study of castration-resistant prostate tumour xenografts in mice, tasquinimod inhibited the formation of lung and lymph node metastases and suppressed the formation of tumours in bone after intratibial implantation [26].

Extensive studies have been undertaken to evaluate the mechanism underlying these anti-tumour effects of tasquinimod, focusing on its effects on the immune system and angiogenesis.

## Immunomodulatory effects of tasquinimod

Infiltrating myeloid cells in the tumour microenvironment are essential for tumour growth, metastasis and angiogenesis [6, 13, 15]. Growing evidence indicates that tasquinimod targets the myeloid cell compartment and modulates local tumour immunity by binding to the calcium-binding protein S100A9 [27].

S100A9 is a multifunctional protein which is often co-expressed with S100A8, another member of the S100 protein family. They are classified as damage-associated molecular pattern (DAMP) molecules that are secreted from myeloid cells upon activation [28]. S100A9 binds in the presence of zinc and calcium to Toll-like receptor 4 (TLR4) and the receptor of advanced glycation end products (RAGE) and promotes pro-inflammatory responses [29, 30]. S100A9 may be involved in cancer progression by several mechanisms. For instance, S100A9 expressed by myeloid cells and tumour cells in the tumour microenvironment is important for the accumulation and activation of regulatory myeloid cells (e.g. MDSC and TAM) [31, 32]. Furthermore, S100A9 also has a role in recruiting both inflammatory cells and tumour cells to metastatic sites [33–35]. Thus, blocking the function of S100A9 by small molecule inhibitors may provide a new approach for the prevention of tumour growth and metastasis [10, 36].

High levels of S100A9 have been found in the microenvironment of several forms of tumours, and a high expression level has been correlated with poor tumour differentiation [37, 38]. In bladder cancer, increased expression of S100A8 and S100A9 proteins is associated with a worse prognosis [39]. Similarly, the expression of S100A9 is increased early in the course of prostate cancer and may contribute to tumour development and metastasis. Moreover, the presence of circulating S100A9 has been suggested as a marker to distinguish prostate cancer from benign prostate enlargement [40].

Tasquinimod binds to S100A9 and inhibits its interaction with receptors such as RAGE and TLR4. The importance of these interactions in tumour development was illustrated in experiments in knock-out mice showing that in the absence of S100A9 or TLR4, the development of spontaneous prostate cancer tumours was delayed [27]. In the EL4 mouse lymphoma model, tasquinimod treatment inhibited tumour growth, which was associated with reduced expression of TGF-β. TGF-β is an immunosuppressive cytokine overexpressed by tumours which attracts MDSC into the tumour stroma and plays an important role in regulating adaptive immune responses [27].

Tasquinimod has shown anti-tumour effects in several other syngeneic tumour models, possibly by modulating the accumulation and function of regulatory myeloid cells, thereby reducing immunosuppression in the tumour microenvironment. Accordingly, the combination of tasquinimod with immunotherapy using tumour-targeted superantigens (TTS) resulted in a significant enhancement of anti-tumour effects and an increase in the number of tumour-infiltrating CD8^+^ T cells in the mouse B16 melanoma model [41]. It is likely that these effects are the result of the changes in infiltrating regulatory myeloid cells induced by tasquinimod. Specifically, tasquinimod reduced the subpopulation of CD11b^+^Ly6C^high^ MDSC and increased the subpopulation of CD11b^+^Ly6G^+^ MDSC, thus shifting the infiltrating myeloid cell population towards a more granulocytic phenotype [41]. Tasquinimod also reduced the accumulation of MDSC in a castration-resistant, syngeneic transplantable murine prostate cancer model (CR Myc-Cap) and enhanced the survivin cancer vaccine efficacy in this model [42]. Further evidence for immunomodulatory effects by tasquinimod was obtained in several syngeneic tumour models, where tasquinimod treatment induced a shift within the F4/80^+^ tumour-infiltrating macrophages from a strong dominance of M2 into a less suppressive M1 myeloid population (unpublished data). Thus, tasquinimod may alter the balance between M1 and M2 macrophages in the tumour microenvironment further promoting anti-tumour effects.

There are many links between the immune system and angiogenic pathways, including the potential for TAM and MDSC to promote angiogenesis through production of pro-inflammatory cytokines and endothelial growth factors [11, 13, 43]. Hence, the effects of tasquinimod on regulatory myeloid cells may, in addition to relieving the immunosuppressive pressure within the tumour, drive the anti-angiogenic effects discussed below, as well as complementing other anti-angiogenic mechanisms.

## Anti-angiogenic effects of tasquinimod

Tasquinimod has been shown to inhibit angiogenesis in a variety of models: it inhibits endothelial cell growth and capillary tube formation from aortic rings in in vitro (ex vivo) models, decreases the density of tumour microvessels (CD31-positive) and reduces real-time tumour blood flow and tumour oxygenation in vivo [22]. However, tasquinimod has no direct effects on VEGF signalling; it does not interact with VEGF or inhibit VEGF receptor tyrosine kinase activity, in contrast to existing approved anti-angiogenic agents such as bevacizumab and VEGF receptor tyrosine kinase inhibitors. The evidence suggests that the effects of tasquinimod are mediated through down-regulation of HIF-1α-controlled genes, such as VEGF, CXCR4, lysyl oxidase (LOX) and SDF-1 [26, 44].

Several mechanisms may underlie the inhibitory effects of tasquinimod on the angiogenic switch. As already discussed, the inhibitory effects of tasquinimod on myeloid cells (MDSC and TAM) also have the potential to inhibit angiogenesis. A recent original research report indicates that another possible mechanism for the effect of tasquinimod on HIF-1-controlled genes may be interaction with histone deacetylases (HDAC). Histone modifications such as acetylation are involved in chromatin alterations and tumorigenesis [45]. HDAC function is closely involved in angiogenesis and tumour progression through control of hypoxia-responsive genes, and HDAC inhibitors have the potential to inhibit angiogenesis, by altering the balance between pro- and anti-angiogenic factors [46–49]. Tasquinimod locks HDAC4 in an inactive configuration by binding allosterically and with high affinity to its zinc-binding domain. This inhibits formation of the HDAC4/HDAC3/nuclear co-receptor (NCoR) repressor complex [50], without inhibiting enzyme activity. This affects downstream HDAC-mediated deacetylation of targets including HIF-1α [51, 52] and blocks activation of the HIF-1α-p300 complex, with a subsequent reduction in HIF-1α-mediated target gene expression [50].

Tasquinimod also increases tumour levels of TSP-1 [44], an endogenous anti-angiogenic agent that has been shown to promote the recruitment of M1 polarised TAM, which inhibit angiogenesis and tumour progression, relative to the pro-angiogenic M2 phenotype [53]. Consequently, tasquinimod-driven increases of TSP-1 in the tumour may alter the balance between M1 and M2, favouring anti-angiogenesis [44]. In support of this, tasquinimod has been shown to reduce the frequency of CD206^+^M2 TAM in two mouse models [41, 54, 55].

## Medical need and role of tasquinimod

The tumour microenvironment promotes the growth and spread of many types of solid tumours through suppression of immune responses and offers an important opportunity for therapeutic intervention, as already discussed. Targeting the myeloid cell compartment is a novel approach that has a vast potential for treatment for many different forms of cancer. Tasquinimod is a leading drug that demonstrated potent effects on modulating the tumour cell microenvironment in several preclinical models leading to extensive clinical evaluations in various types of tumours including prostate cancer. A proof of concept study is ongoing to investigate the clinical activity of tasquinimod in other tumour types, including several where there is a rationale to target MDSC and in which there is already evidence of benefit with anti-angiogenic therapy: advanced or metastatic hepatocellular carcinoma, ovarian carcinoma, renal cell carcinoma and gastric carcinoma after progression with standard therapies (NCT01743469). This trial follows a novel design in which the endpoint is the proportion of patients who have neither progressed (RECIST and Choi criteria [56]) nor died at specified time points in each cohort.

Tasquinimod has already shown promising effects in the treatment of prostate cancer in clinical studies. There is also encouraging preclinical evidence that tasquinimod can enhance the effects of other anti-neoplastic treatments, including radiotherapy, chemotherapy, androgen ablation and immunotherapy. Considering that inhibitory effects of MDSC on innate anti-tumour immunity represent a significant barrier to cancer immunotherapy, there emerged a strong rationale for combining tasquinimod with other immunological approaches, supported by preclinical work discussed earlier including the combination with immunotherapy using TTS and with a survivin cancer vaccine [41, 42, 55]. In addition, it has been proposed that emergence of resistance to VEGF receptor tyrosine kinase inhibitors may be mediated in part by the survival of MDSC, which provide sustained angiogenic drive as well as immunosuppression [57]. Hence, in view of its toxicity profile and its effects on MDSC and HIF-induced angiogenesis, there are several experiments to support the use of tasquinimod either in combination with anti-VEGF drugs in the first-line setting or after failure of conventional anti-angiogenic agents in patients with recurrent clear-cell renal cell carcinoma or hepatic cancer.

Tasquinimod significantly increased the effects of fractionated radiation on endothelial and prostate cancer cells in vitro and in vivo [25]. Based on the effects on tumour oxygenation, vascular volume and blood vessel density, this was attributed to the inhibition by tasquinimod of the angiogenic rebound caused by fractionated radiation.

Preclinical results indicate that the anti-cancer effects of tasquinimod are enhanced when it is combined with taxanes [24], and a Phase I trial is currently evaluating the combination of tasquinimod and cabazitaxel in metastatic castration-resistant prostate cancer (CRPC) patients after failure of docetaxel (NCT01513733).

The combination of tasquinimod with androgen ablation is also effective in animal models [24]. The synergistic effects of tasquinimod combined with androgen ablation may be relying in part on angiogenesis, although possible effects on the androgen axis cannot be ruled out. Androgen ablation results in a temporary anti-angiogenic effect as a consequence of up-regulation of TSP-1, which is subsequently overcome by increased VEGF production [58]. In addition, as CXCR4 may promote AR signalling in the absence of hormone [59], a further role for combination with anti-androgens can be envisaged for tasquinimod.

There is also the potential to combine tasquinimod with newer treatments for metastatic CRPC that address the continued dependence on androgen signalling such as abiraterone, an androgen synthesis inhibitor, and enzalutamide, a potent AR antagonist. The need for more treatment options is highlighted by the recent report that only very modest responses to abiraterone were seen in tumours escaping enzalutamide treatment [60]; given its different mode of action from androgen-directed therapies, tasquinimod could be valuable in this situation [57].

## Discussion and conclusions

**Fig. 1:**
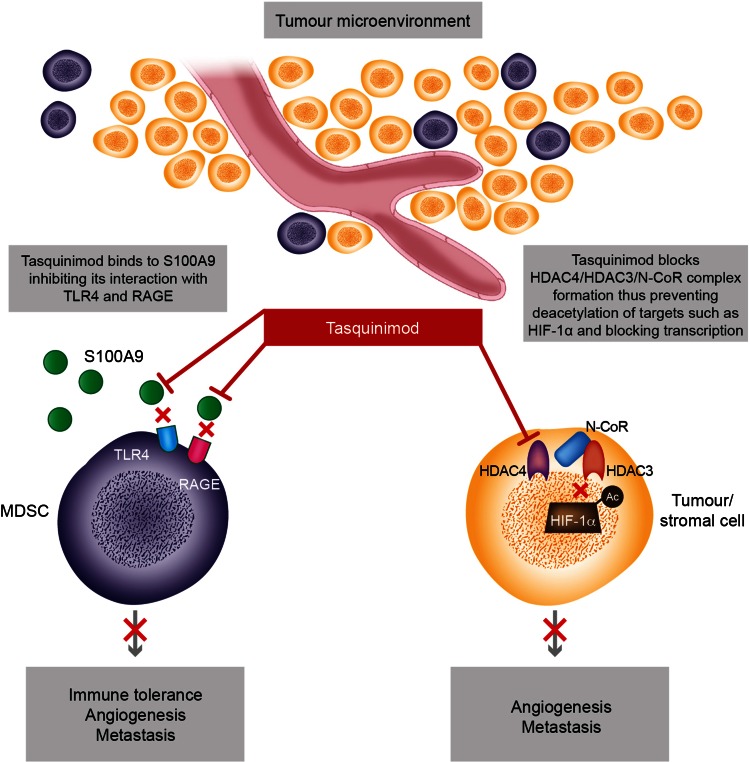
Overview of tasquinimod mode of action

Tasquinimod targets the tumour microenvironment with the potential to overcome tumour-associated immunosuppression and inhibit angiogenesis, metastasis and tumour growth (Fig. 1). It binds to the S100A9 protein, a calcium-binding protein involved in inflammatory events and cancer development, inhibiting its interaction with TLR4 and RAGE. This reduces the infiltration of MDSC, influences the balance between M1 and M2 TAM and enhances anti-tumour immune responses. The mechanisms underlying the anti-angiogenic effects of tasquinimod are not fully understood, but do not involve direct effects on VEGF or VEGF receptor tyrosine kinase inhibitors. Inhibition of angiogenesis may be a further consequence of MDSC inhibition, while selective allosteric modulation of HDAC4 has also been proposed to mediate blocking of the angiogenic switch. Through its direct and indirect effects, tasquinimod has the potential to modify immunomodulatory and angiogenic pathways leading to an anti-cancer treatment effect. These complementary effects on the tumour microenvironment are the subject of ongoing evaluation which is likely to provide further insights into its unique mode of action.

Tasquinimod is a promising first-in-class anti-cancer agent with a unique combination of effects on the tumour microenvironment which is currently undergoing clinical evaluation as an oral once-daily cancer treatment.

Metastatic CRPC is an area of significant unmet clinical need and the initial focus for the clinical development of tasquinimod. In a randomised, Phase II, double-blind, placebo-controlled trial in men with minimally symptomatic metastatic CRPC, 69 % of tasquinimod-treated patients were progression-free at 6 months compared with 37 % of the placebo group (*p* < 0.001), and median progression-free survival was increased from 3.3 to 7.6 months (*p* = 0.0042), reflecting a significant delay in disease progression with tasquinimod treatment [61]. Further support for an effect on the tumour microenvironment was provided by the effects on biomarkers such as bone alkaline phosphatase, VEGF and PSA. On the basis of these results, a Phase III trial is ongoing in a similar patient population to confirm the effects of tasquinimod in delaying progression and prolonging survival; progression-free survival is the primary endpoint and the trial is powered to show an effect on overall survival (NCT01234311). A Phase II trial is investigating the potential of tasquinimod as maintenance therapy in metastatic CRPC not progressing after chemotherapy with docetaxel (NCT01732549). The encouraging results already seen in the Phase II trial support the further evaluation of tasquinimod in prostate cancer, despite the lack of effect of established anti-angiogenic agents, including bevacizumab and sunitinib, on survival in randomised trials. The benefits of tasquinimod may be attributed to its broad pleiotropic effects on the tumour microenvironment, including a high capacity to overcome tumour-related immunosuppression.

In summary, based on its mechanism of action, tasquinimod represents a novel targeted therapy with potential in many tumour types, and proof-of-concept trials are ongoing; it may also be suitable for combination strategies in different solid tumours. Further preclinical and clinical testing will fully exploit its therapeutic potential as a novel agent targeting the tumour microenvironment rather than tumour cells directly.
